# Missed Opportunities for Sedation and Pain Management at a Level III Neonatal Intensive Care Unit, India

**DOI:** 10.3389/fped.2016.00007

**Published:** 2016-02-23

**Authors:** Shikha Y. Kothari, Ashish R. Dongara, Somashekhar M. Nimbalkar, Ajay G. Phatak, Archana S. Nimbalkar

**Affiliations:** ^1^Department of Pediatrics, Pramukhswami Medical College, Karamsad-Anand, India; ^2^Central Research Services, Charutar Arogya Mandal, Karamsad-Anand, India; ^3^Department of Physiology, Pramukhswami Medical College, Karamsad-Anand, India

**Keywords:** neonatal pain, opportunities, nurse, neonatal intensive care, critical care

## Abstract

**Background:**

Neonates in the neonatal intensive care unit (NICU) undergo a multitude of painful and stressful procedures during the first days of life. Stress from this pain can lead to neurodevelopmental problems that manifest in later childhood and should be prevented.

**Objective:**

To determine the number of painful procedures performed per day for each neonate, to verify documentation of painful procedures performed, and to, subsequently, note missed opportunities for providing pain relief to neonates.

**Methods:**

We conducted a cross-sectional study at a level III NICU located in a rural part of western India. A total of 69 neonates admitted for more than 24 h were included. Twenty-nine neonates were directly observed for a total of 24 h each, and another 40 neonatal records were retrospectively reviewed for the neonate’s first 7 days of admission. All stressful and painful procedures performed on the neonate were recorded. Also recorded were any pharmaceutical pain relief agents or central nervous system depressants administered to the neonate before or at the time of the procedures. Average nurse–patient ratio was also calculated. Data were analyzed using descriptive statistics.

**Results:**

A documentation deficit of 2.2% was observed. The average nurse–patient ratio was 1.53:1. A total of 13711 procedures were recorded, yielding 44.1 (38.1 stressful, 3.8 mildly painful, and 2.2 moderately painful) procedures per patient day. Common stressful procedures were position changing (2501) and temperature recording (2208). Common mildly and moderately painful procedures were heel prick (757) and endotracheal suctioning (526), respectively. Use of pharmacological agents coincided with 33.48% of the procedures. The choice of drug and time of administration were inappropriate, indicating that the pharmacological agents were intended not for pain relief but rather for a coexisting pathology or as sedation from ventilation with no analgesia.

**Conclusion:**

Stressful procedures are common in the NICU; mildly and moderately painful procedures fairly common. Almost two-thirds of the times, no pharmaceutical pain relief methods were used, and when administered, the pharmaceutical agents were seldom intended for pain relief; this implies poor pain management practices and emphasizes the imperative need for educating NICU nurses, residents, fellows, and attendings.

## Introduction

The International Association for the Study of Pain (IASP) defines pain as “an unpleasant sensory and emotional experience associated with actual or potential tissue damage, or described in terms of such damage” (1). This definition is not applicable for certain population groups like newborns (2). Pain in newborns is a highly complex phenomenon that needs to be well understood by health-care personnel involved in neonatal care (3, 4).

The past two decades have seen an increase in knowledge regarding the physiology of neonatal pain, the development of multiple valid pain assessment tools, and the formulation of various guidelines for pain management in newborns. However, discrepancies and shortcomings in neonatal pain assessment and management still exist among various neonatal care units (5–7).

Neonates repeatedly exposed to pain tend to perceive pain as more severe (8). Such pain can lead to an alteration in the neonate’s nociceptive circuitry in adulthood (9). There can be long-term neurodevelopmental, emotional abnormalities, and social-functional abnormalities (10).This alteration depends on the type and intensity of pain experienced (11). Preterm neonates have a lower threshold for pain and are therefore more affected by pain (12, 13). These factors need to be taken into consideration when neonatologists and neonatal intensive care unit (NICU) staff establish a management plan.

Painful and stressful procedures are an inevitable side effect of clinical management. A majority of neonates in the NICU undergo a multitude of painful and stressful procedures during the first few days of life (14, 15). Epidemiological studies have demonstrated that the pain induced by these procedures is neither identified properly nor adequately treated (16).

Past studies have demonstrated the importance of developing established, evidence-based prevention and treatment protocols for neonatal pain (17–19) and the benefits of incorporating pain management as a routine component of care provided to “all neonates, regardless of their gestational age or severity of illness” (17). In 2006, the American Academy of Pediatrics and the Canadian Pediatric Society collaborated to formulate guidelines recommending implementation of a “pain-prevention program” in every health-care facility providing care to neonates. According to these guidelines, such a program should include “strategies for routinely assessing pain, minimizing the number of painful procedures performed, effectively using pharmacologic and non-pharmacologic therapies for the prevention of pain associated with routine minor procedures, and eliminating pain associated with surgery and other major procedures” (20).

However, while such guidelines and recommendations have been in existence for nearly a decade in developed countries, neonatal pain and its prevention and management is still in its evolving phase in developing nations. Though National Neonatology Forum (NNF) of India established guidelines regarding neonatal procedural pain and its management a few years ago (13), there is insufficient data available regarding the frequency of procedural exposure, the identification and/or the prevention and management of pain in neonates admitted to the NICU (21, 22). Simple pharmacological and non-pharmacological methods that reduce pain are not practiced. Previous studies show that NICU staff lack knowledge about neonatal pain, and that their attitudes hinder pain management in neonates (22, 23).

### Goals and Objectives

The present study sought to determine the number of painful procedures performed per day for each neonate to verify the documentation of painful procedures performed and to, subsequently, note the number of missed opportunities for providing pain relief to neonates at an NICU in western India.

## Materials and Methods

### Setting

We conducted a cross-sectional observational study at the level III NICU of Shri Krishna Hospital in Karamsad, which is a rural village located in the state of Gujarat, in western India.

### Definitions

A procedure was defined as “a sporadic medical, nursing or surgical, and diagnostic or therapeutic activity performed on the child” (14). This definition implies that a continuous therapeutic procedure could not be counted as a procedure, e.g., ventilation. In the absence of an appropriate/accurate definition of neonatal pain, we chose to use the IASP definition of pain (1). A procedure was considered painful if it “invaded the neonate’s bodily integrity, causing skin injury or mucosal injury from the introduction or removal of foreign material into the airway or the digestive or urinary tract” (14). A procedure was considered to be stressful when it “disturbed the equilibrium that was existent between the neonate and its environment or annoyed the neonate” (14). All of the procedures were classified as either stressful or mildly, moderately, or severely painful, as described by the NNF Clinical Practice Guidelines (13). A panel of four neonatologists reviewed the NNF procedural pain classification and tailored it to the present study. Handling by caregivers was considered stressful. Maternal touch was not deemed to be stressful.

### Subject Recruitment and Study Procedure

The study was conducted in two phases. The first phase was an observational study of 29 neonates. The purpose of this phase was to determine the accuracy of documentation. Only neonates admitted for more than 1 day were included in this phase. The second phase comprised a retrospective assessment of the case records of 40 neonates. The first 7 days of NICU admission were reviewed from the neonatal case records. All of the documented stressful and painful procedures were recorded in a predesigned *pro forma* for each neonate. The same file audit method was used in both phases.

### First Phase

In the first phase, one of the authors directly observed 29 neonates in the NICU for a total of 738 patient h over 17 days. The number of painful procedures documented in each neonate’s respective case record was compared to the number of procedures directly observed. Pain relief measures were also recorded and compared to the documented data.

Our NICU is so structured that an observer in the NICU can monitor activities in four to five neonates at a time. Twenty-nine infants were observed for 24 h each. The observation was divided into blocks of 4–6 h spread over 2–3 days. Once 24 h of observation per neonate was completed, that particular neonate was not included further for data collection. No fixed timings were determined for observation; time slots were chosen randomly. However, we ensured that all periods of the day and night were covered to minimize bias. Average nurse–patient ratio was also calculated, by averaging the ratios from each of the various time slots during which the neonates were observed.

The neonates’ case records were then reviewed for documentation, and a documentation deficit was calculated based on the actual documentation of procedures by the investigators versus the entries done by the NICU staff in the patient records. The documentation check was done after the live data collection was completed, in the hospital medical records department.

### Second Phase

Neonatal intensive care unit admissions data was retrieved from the hospital medical records department. Neonates having admission for more than 7 days were carved out from the data. Then, using the random number generation feature of Microsoft Excel, 40 files were selected for review.

Both nursing notes and resident/doctor notes, along with other documentation in the case records like investigation sheets and drug charts, were reviewed to determine the number of procedures performed and to determine the number of instances during which pharmaceutical pain relief agents, and/or central nervous system depressants (barbiturates, benzodiazepines), were administered before or at the time of the procedures. The specific drug administered, the drug’s known duration of action, time of drug administration, and time and length of the procedure were all taken into consideration when determining whether a central nervous or nociceptive depressant action was in effect for the duration of each procedure. This was done during the first phase as well.

The documentation deficit from the first phase was used to calculate projected numbers of procedures occurring each day for these 40 neonates.

### Ethics Approval

The institutional Human Research and Ethics Committee (HREC) approved the study and also duly approved a waiver of written informed consent.

### Data Analysis

The data was entered into Microsoft Excel. It was then analyzed using a combination of Microsoft Excel and Stata 14, with descriptive statistics.

## Results

The study included 69 neonates, 29 of whom were directly observed in the NICU for a total of 738 patient h, and 40 of which whose patient records were retrospectively studied for the first 7 days of admission in the NICU. In the first phase, 2121 procedures were observed, of which 2075 were noted in their respective neonates’ case records. There was, therefore, a deficit in documentation for 2.2% of the procedures. Considering this, the authors decided to ignore the minor deficit in documentation and decided to analyze all 69 of the patient records together. On direct observation of the 29 neonates, it was observed that the average patient–nurse ratio was 1.53:1 at the center where the present study was conducted.

A majority of the 69 neonates were male, preterm, born by Cesarean section, and had low birth weight and poor APGAR score at 1 min after birth. A large proportion developed early onset sepsis. Respiratory distress and hypoxic ischemic encephalopathy were also quite common. A large proportion required invasive or non-invasive airway support and long stay in the NICU. Nearly half of the neonates had abnormal neurology at the time of admission. See Table 1 for sociodemographic and clinical profile of the neonates, and Table 2 for common clinical diagnoses.

**Table 1:** **Sociodemographic and clinical profile of neonates**.

	29 observed neonates			40 file audits			All 69 neonates		
	Pain relief	Non-pain relief	Total	Pain relief	Non-pain relief	Total	Pain relief	Non-pain relief	Total
**Sex**									
Male	9	10	19	12	15	27	21	25	46
Female	3	7	10	3	10	13	6	17	23
**Place of birth**									
Outborn	7	9	16	6	6	12	13	15	28
Inborn	5	8	13	9	19	28	14	27	41
**Birth weight (kg)**									
Mean (SD)	2.062 (0.8377)	1.646 (0.6438)	1.818 (0.7457)	2.222 (0.5427)	1.924 (0.5311)	2.036 (0.5480)	2.151 (0.6798)	1.812 (0.5883)	1.944 (0.6428)
Normal (≥2.5 kg)	5	3	8	6	5	11	11	8	19
LBW (<2.5 kg)	3	8	11	7	16	23	10	24	34
VLBW (<1.5 kg)	3	4	7	2	4	6	5	8	13
ELBW (<1 kg)	1	2	3	0	0	0	1	2	3
**Gestational age (weeks)**									
Mean (SD)	35.5 (4.10)	34.5 (3.99)	34.9 (3.99)	36.3 (2.99)	35.5 (2.63)	35.8 (3.76)	35.9 (3.47)	35.1 (3.24)	35.4 (3.33)
**Full term**									
(≥37 weeks)	6	6	12	6	7	13	12	13	25
**Late preterm**									
(34–36 weeks)	2	3	5	6	14	20	8	17	25
**Early preterm**									
(32–34 weeks)	1	3	4	2	2	4	3	5	8
(30–32 weeks)	1	3	4	1	1	2	2	4	6
(28–30 weeks)	2	1	3	0	1	1	2	2	4
(26–28 weeks)	0	1	1	0	0	0	0	1	1
**Gestational maturity**									
AGA	8	5	13	8	14	22	16	19	35
SGA	4	12	16	7	11	18	11	23	34
**Head circumference**									
Mean (SD)	31.2 (4.05)	28.8 (3.26)	29.8 (3.75)	31.7 (2.03)	30.4 (2.65)	30.9 (2.49)	31.5 (3.04)	29.8 (2.99)	30.4 (3.11)
**Length**									
Mean (SD)	45.4 (4.64)	42.0 (4.70)	43.4 (4.90)	45.4 (3.58)	43.3 (4.61)	44.2 (4.33)	45.4 (4.00)	42.8 (4.64)	43.8 (4.56)
**Mode of birth**									
Spontaneous vaginal delivery	6	11	17	5	6	11	11	17	28
LSCS under spinal anesthesia	5	5	10	6	12	18	11	17	28
LSCS under general anesthesia	0	1	1	2	7	9	2	8	10
Assisted vaginal delivery (vacuum)	1	0	1	0	0	0	1	0	1
Assisted vaginal delivery (forceps)	0	0	0	2	0	2	2	0	2
**Liquor**									
Clear	4	7	11	7	14	21	11	21	32
Meconium stained	2	2	4	5	7	12	7	9	16
Not known	6	8	14	3	4	7	9	12	21
**Cried immediately after birth**									
Cried immediately	5	12	17	5	7	12	10	19	29
Weak cry	1	2	3	3	5	8	4	7	11
Did not cry immediately	6	3	9	7	13	20	13	16	29
**Bag/mask required**									
Yes	8	4	12	11	16	27	19	20	39
No	4	13	17	4	9	13	8	22	30
**APGAR score**									
After 1 min [mean (SD)]	4.4 (1.52)	5.5 (1.93)	5.08 (1.80)	4.6 (1.94)	4.62 (2.22)	4.60 (2.11)	4.5 (1.74)	4.86 (2.15)	4.74 (2.01)
After 5 min [mean (SD)]	6.4 (2.07)	8.13 (1.36)	7.46 (1.81)	7.0 (2.35)	7.24 (1.67)	7.17 (1.86)	6.8 (2.2)	7.48 (1.62)	7.26 (1.83)
After 10 min [mean (SD)]	6.0 (1.41)	8.00 (0.0)	7.00 (1.41)	7.3 (2.31)	7.71 (1.38)	7.60 (1.58)	6.8 (1.9)	7.78 (1.20)	7.43 (1.50)
**Maternal high risk factors present**									
Yes	9	15	24	14	23	37	23	38	61
No	3	2	5	1	2	3	4	4	8
**Neurological status**									
Good	2	11	13	8	17	25	10	28	38
Poor	10	6	16	7	8	15	17	14	31
**Age on admission (number of days)**									
Mean (SD)	1.42 (0.793)	1.53 (1.23)	1.48 (1.06)	1.40 (1.056)	2.56 (5.66)	2.13 (4.52)	1.41 (0.931)	2.14 (4.43)	1.86 (3.50)
**Age on discharge (number of days)**									
Mean (SD)	14.4 (17.1)	11.0 (9.25)	12.4 (12.9)	15.1 (9.82)	15.0 (9.13)	15.1 (9.27)	14.8 (13.3)	13.4 (9.28)	14.0 (10.9)
**Duration of stay (days)**									
Mean (SD)	14.0 (17.2)	10.5 (9.18)	11.9 (13.0)	14.8 (9.92)	13.5 (7.82)	14.0 (8.56)	14.4 (13.4)	12.3 (8.42)	13.1 (10.6)
**Nil by mouth**									
Yes	9	3	12	8	4	12	17	7	24
No	3	14	17	7	21	28	10	35	45

*“Pain relief” neonates are those receiving pharmaceutical agents for at least one procedure. “Non-pain relief” neonates have not received any pharmaceutical pain relief/central nervous system depressing agents. LSCS, lower segment Cesarean section*.

**Table 2:** **Common clinical diagnoses**.

29 observed patients (frequency)	40 file audits (frequency)
Early onset sepsis (21)	Early onset sepsis (21)
Respiratory distress syndrome (12)	Respiratory distress syndrome (14)
Hyperbilirubinemia (7)	Meconium stained liquor (11)
Apnea of prematurity (5)	On ventilatory support (11)
On ventilatory support (5)	Birth asphyxia (8)

Overall, a total number of 13711 procedures were recorded. This amounted to 44.1 procedures per patient day. Of these 44.1 procedures per patient day, 38.1 were stressful procedures, 3.8 were mildly painful, and 2.2 were moderately painful procedures. Please see Table 3 for the specifics of each phase.

**Table 3:** **Procedure documentation data**.

	Observed patients and files (29)		File audits (40)	
	Observed	Documented	Documented	Projected
Total number of procedures	2121	2075	11636	11892
Procedures per patient h	2.87	2.81	1.73	1.77
Stressful procedures	1705	1691	10170	10394
Stressful procedures/patient h	2.31	2.29	1.51	1.52
Mildly painful procedures	219	195	985	1007
Mildly painful procedures/patient h	0.3	0.26	0.15	0.17
Moderately painful procedures	197	189	481	492
Moderately painful procedures/patient h	0.27	0.26	0.07	0.07

Table 4 depicts the common procedures observed and documented. The most common stressful, mildly painful, and moderately painful procedures were position-changing, heel prick for blood sugar testing, and endotracheal suctioning, respectively. Severely painful procedures entail surgical procedures, which were not encountered in any of these neonates.

**Table 4:** **Common procedures**.

	29 observed patients	40 file audits
Most common stressful procedures (frequency)	Position change (311)	Position Change (2190)
	Temperature (283)	Temperature (1925)
	Abdominal girth (280)	Abdominal girth (1888)
	Diaper change (280)	Diaper change (1795)
	Physical exam (278)	Physical exam (1340)
Most common mildly painful procedures (frequency)	Heel prick (130)	Heel prick (627)
	Venipuncture (27)	Blood sample (143)
	Blood sample (18)	Venipuncture (62)
	NT insertion (14)	Umbilical catheterization (62)
	Umbilical catheterization (7)	IV removal (25)
Most common moderately painful procedures (frequency)	Suction (178)	Suction (351)
	Lumbar puncture (5)	Remove ET (19)
	Change umbilical dressing (4)	Change umbilical dressing (23)
	ET adjust (3)	ET insertion (30)
	Remove umbilical dressing (2)	Remove umbilical dressing (37)

*NT, nasogastric tube; ET, endotracheal tube*.

**Figure 1:**
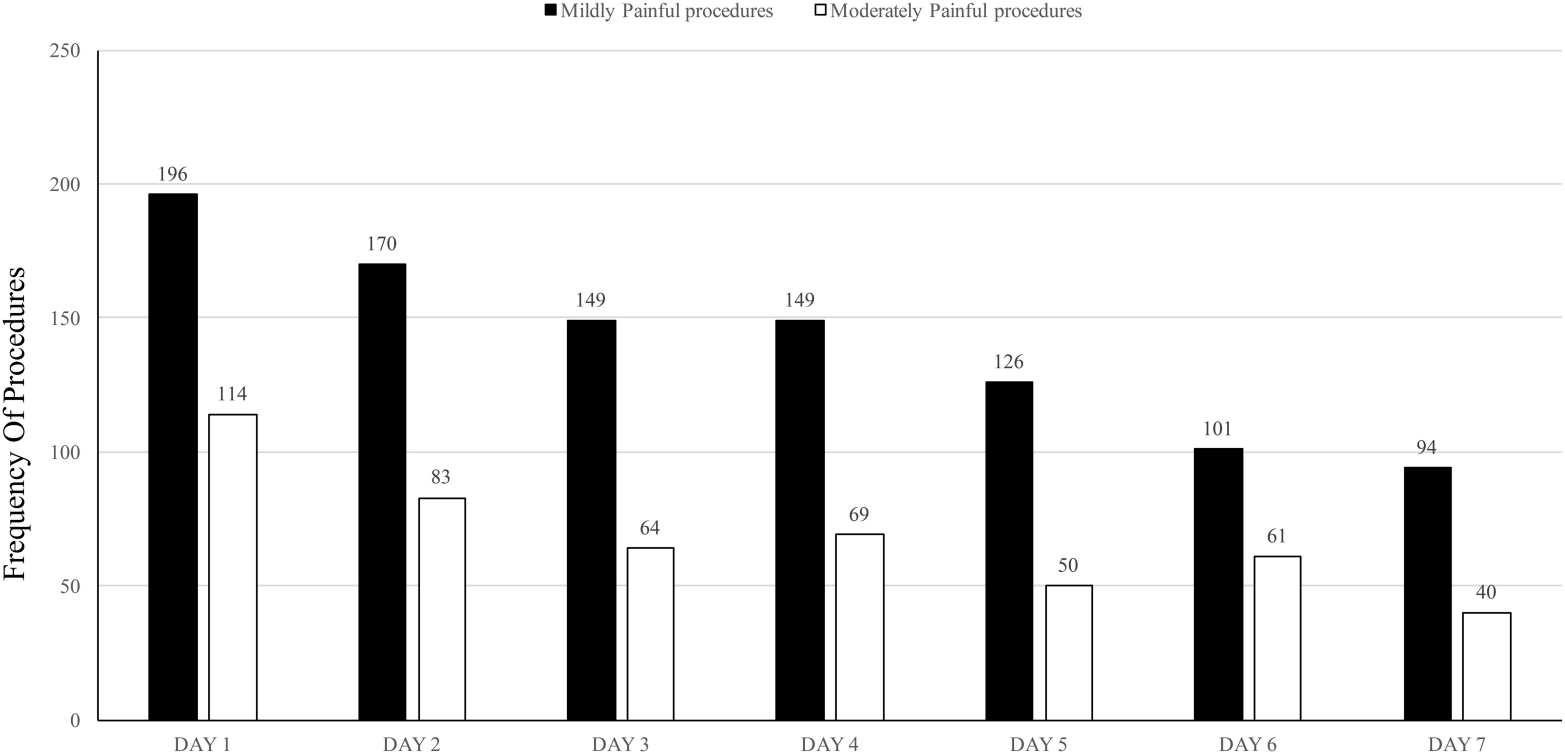
**Distribution of painful procedures over the first 7 days of admission**.

A majority of painful procedures occurred over the first few days of admission. There was a general trend of decreasing numbers of painful procedures as the duration of admission progressed. Such a trend has not been observed for the stressful procedures (Figure 1).

**Figure 2:**
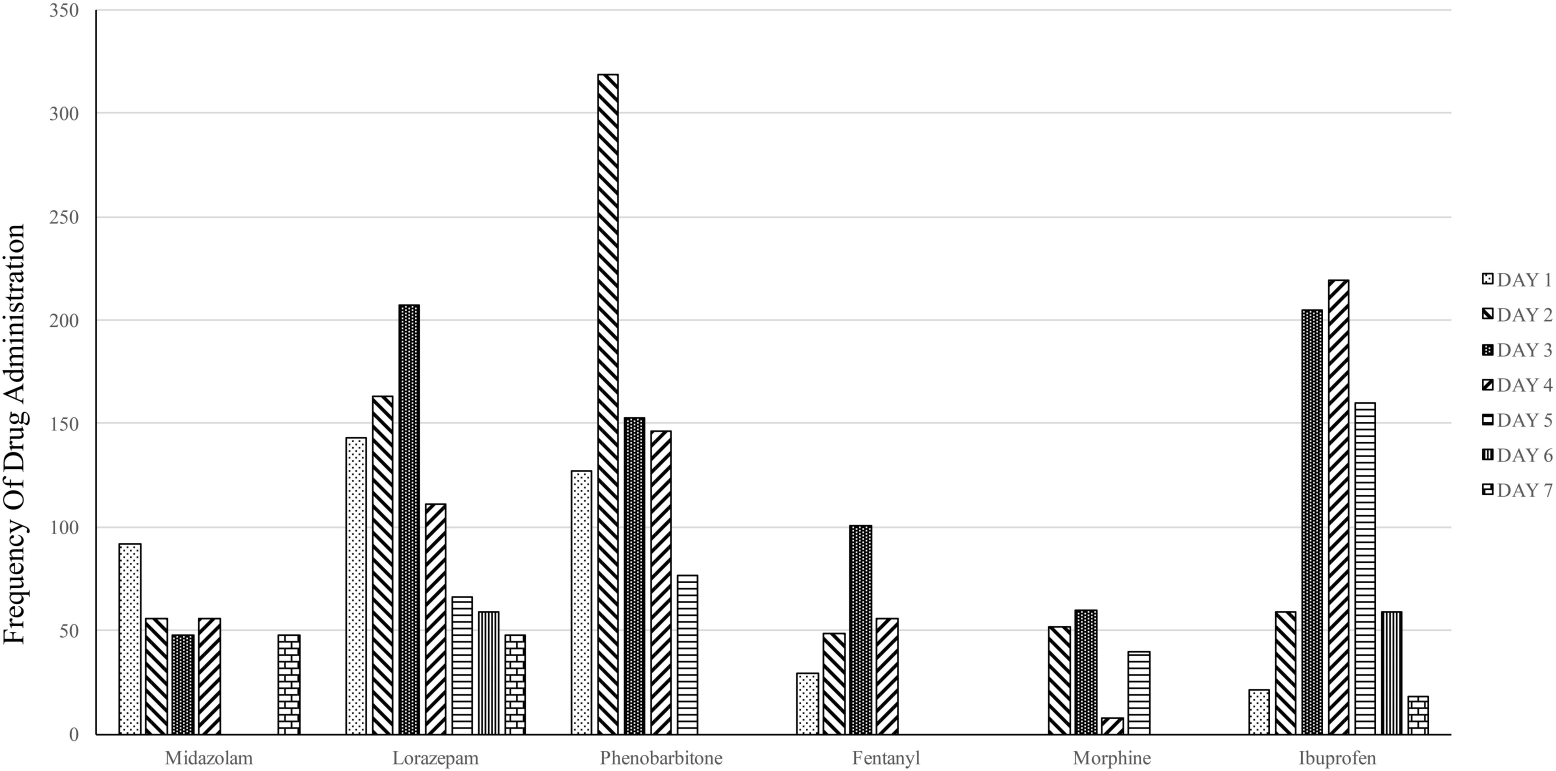
**Distribution of pharmaceutical pain relief agents over first 7 days of admission**.

A total of 27 (39.1%) out of 69 neonates received some pharmaceutical agent for pain relief/sedation/central nervous system depression. Such pharmaceutical agents were used for 4592 (33.5%) of the procedures. Lorazepam (1169; 25.5%), phenobarbitone (1145; 24.9%), ibuprofen (741; 16.1%), midazolam (568; 12.4%), fentanyl (495; 10.8%) and morphine (242; 5.3%) were the commonly used pharmacological agents (Figure 2). The use of pharmacological agents did not correlate with the incidence of painful procedures.

## Discussion

A total of 44.1 procedures were recorded per patient day. There were 3.8 mildly painful procedures and 2.2 moderately painful procedures per patient day; these are significant numbers and imply that the neonates undergo a very high level of daily stress, which can have adverse effects on their health and neurodevelopment in the near future (Table 2). The first 7 days of admission were reviewed because the highest number of NICU interventions occurs for neonates weighing >1000 g, which comprise a large proportion of our admissions. An important observation was that there was a decrease in the incidence of painful procedures as the stay of the neonate progressed (Figure 1). This reflects upon the fact that neonates require most procedures on the first day of admission and that they tend to stabilize subsequently and therefore require less painful interventions. It also reflects the seriousness and subsequent gradual improvement of the neonates’ general condition and suggests that this is the peak time for implementing effective pain relief measures to ensure adequate pain management.

Other studies have also recorded a similarly high number of stressful/painful procedures in the NICU (14, 22). We found position changing, abdominal girth measurement, temperature recording, diaper changing, and physical examination were the most common stressful procedures (Table 3). This is similar to other studies (21, 22). Such stressful/painful procedures always occur as a part of neonatal intensive care and are inevitable (21). Clubbing of these procedures is a routine practice at the study center. Minimizing unnecessary procedures, and practicing evidence-based medicine, are the key to easing pain in these neonates.

Non-pharmacological measures of pain relief have been proven effective for pain relief during stressful and mildly to moderately painful procedures (13, 24–32). Kangaroo mother care, clubbing of procedures at time of breast milk feeding, non-nutritive sucking, swaddling, and oral sweet tasting solutions are regular practices at the center.

Pharmacological measures of pain relief, such as non-steroidal anti-inflammatory drugs (NSAIDs) or opioids/anti-convulsants/sedatives/other central nervous system depressants that partially or completely depress nociceptive pathways, for such short term and mild to moderate procedural pain, are not recommended on account of their poor efficacy and the encountered side effects (33–35). Such measures were used only one-third of the time. The drugs used were inappropriate and, as we believe, were at many times not intended for pain relief. They were instead administered either as sedation for ventilation or were intended for another underlying pathology altogether, such as convulsions or congenital heart disease. As observed in Figure 2, the frequency of pharmacological agent distribution does not correlate with the frequency of painful procedures. This also indirectly indicates that the pharmacological agents were not intended for pain relief. A majority of neonates on ventilator support were only administered barbiturates like phenobarbitone, with no supplementary analgesics. Barbiturates do not provide analgesia; rather, they only provide sedation, anxiolysis, muscle relaxation and amnesia and may even suppress clinical signs of neonatal pain (36). These neonates were therefore under a high amount of unnoticed pain/stress, leading to possible neurological insult with future repercussions in childhood.

Thirty-one patients (44.9%) were neurologically abnormal at the time of admission. Nursing staff may be misled into believing that absence of response in these neonates implies absence of pain. More than 50% of the neonates were born by Cesarean section. Studies demonstrate a dampened pain response in neonates during and up to several hours after vaginal delivery due to catecholamine surge and sympathoadrenal activation, which is absent in neonates born by Cesarean section (37). The importance of this is that neonates born by Cesarean section have a lower threshold for pain than vaginally born neonates.

The nursing staff in the present study documented a majority of the invasive and non-invasive procedures. This is a reassuring fact, indirectly indicating that the staff was sensitive even toward stressful procedures. The nurse–patient ratio at the study center is better than most centers in India (38). Therefore, the authors are of the opinion that this finding regarding documentation practices cannot be generalized to all NICU’s in India, as most of them are not as well staffed (39).

Previous studies conducted at the present institute reflected upon the poor knowledge and attitudes of nurses toward pain (40, 41). The present study reflects on their poor pain management practices. This not only depicts the developing country scenario; it epitomizes the developed country scenario as well (42–44).

Our study design had inherent limitations. The directly observed study of 29 patients was not blinded. Nurses were only informed that the number of procedures was being observed; they were not informed that their documentation would be assessed. Even the mere presence of an observer can result in a falsely improved performance; therefore, it can falsely depict increased documentation. The second part of the study depends on retrospective assessment of records. General timings of drug administration and timings of procedures were documented and, accordingly, correlated; however, it was not feasible to document the exact time of occurrence of the procedure and correlate it with the exact duration of the administered drug’s effect. We also could not document the indication for the medication administration. Use of non-pharmacological methods of pain relief was not documented and therefore not included in the observations. Limitations notwithstanding the study throws up important findings, which, if addressed, can improve the quality of neonatal care.

Interventions targeting nurses’ knowledge, attitudes, and practices regarding neonatal pain need to be undertaken. Doctors and residents, too, should have increased interest in neonatal pain, as without them it is nearly impossible to create a minimal pain environment for neonates (45). Studies conducted previously state that the identification of missed opportunities and utilization of appropriate interventions and implementation of treatment protocols can improve health care substantially (46).

## Conclusion

Neonatal pain is a fairly common entity in the NICU, and appropriate identification and management is a necessity. Stressful procedures are very common in the NICU, and mildly and moderately painful procedures are fairly common. Proper and adequate pain relief measures are seldom used. There is an urgent need for sensitization and education of all personnel involved in neonatal care. Nurses, being the primary caregivers, are the mainstay for addressing this issue; therefore, neonatal pain should be incorporated into routine nursing curriculum. Improved education of the doctors and residents caring for neonates is also of utmost importance, so that unnecessary painful interventions are avoided and proper prevention and treatment protocols for neonatal pain can be established, thereby creating a minimal pain environment for neonates.

## Author Contributions

SK contributed to the design of the study, data acquisition, data analysis, revision of the manuscript for important intellectual content, and final approval of this manuscript. AD contributed to the design of the study, data analysis, writing the manuscript, and final approval of the manuscript. SN contributed to the design and planning of the study, data analysis, revision of the manuscript for important intellectual content, and final approval of this manuscript. AP contributed to study design, data analysis, and revision of the manuscript for important intellectual content and final approval of manuscript. AN contributed to data acquisition, revision of the manuscript for important intellectual content, and final approval of manuscript. All authors agree to be accountable to all aspects of the work, and SN will be the guarantor for the paper.

## Conflict of Interest Statement

The authors declare that the research was conducted in the absence of any commercial or financial relationships that could be construed as a potential conflict of interest.
