# Supplementary material for: Missed Opportunities for Sedation and Pain Management at a Level III Neonatal Intensive Care Unit, India
Source: Front Pediatr. 2016 Feb 23;4:7. doi: 10.3389/fped.2016.00007 (PMC4763094; doi:10.3389/fped.2016.00007)
Supplement: Supplementary file 1 [file Data_Sheet_1.pdf]

Supplementary Material

Missed opportunities for sedation and pain management at a level III neonatal intensive care unit, India

Shikha Y. Kothari, Ashish R Dongara, Somashekhar M. Nimbalkar*, Ajay G. Phatak, and Archana S. Nimbalkar

*** Correspondence:** Somashekhar M. Nimbalkar: somu_somu@yahoo.com

1 Supplementary Tables

Supplementary Table 1. Procedure classification and documentation.

	29 Observed Neonates		40 File Audits	
	**Procedures Observed**	**Procedures Documented**	**Procedures Documented**	**Pharmaceutical Pain Relief**
**Stressful/Disturbing**				
Eliciting Activity	3	0	3	3
Gastric Lavage	1	1	4	2
Echocardiography	2	1	6	1
Remove Ink Footprint	2	1	6	1
Nasogastric Tube Aspiration	1	1	7	2
Nebulizer	9	9	8	0
Standing X Ray	1	1	15	3
Ultrasonography	2	2	15	4
Blood Transfusion	3	3	21	8
Bedside Chest X Ray	7	7	32	15
Weight	68	68	283	34
Non Invasive Blood Pressure	173	164	626	223
Physical Exam	278	278	1340	245
Diaper Change	280	280	1795	254
Abdominal Girth	280	280	1888	267
Temperature	283	283	1925	273
Position Change	311	311	2190	316
Surfactant Administration	1	1	6	1
Total Stressful Procedures	1705	1691	10170	1652

**Mildly Painful**				
Intramuscular Injection	1	1	2	0
Nasogastric Tube Adjust	2	0	2	0
Nasogastric Tube Removal	7	1	2	1
IV Adjust	1	0	3	0
Nasogastric Tube Insertion	14	0	5	2
Change IV	1	1	14	6
IV Removal	6	6	19	7
Remove Umbilical Line	3	2	19	6
Umbilical Catheterization	7	7	25	10
Venepuncture	27	27	62	16
Eye Drops	2	2	62	4
Blood Sample	18	18	143	42
Heel Prick	130	130	627	131
Total Mildly Painful Procedures	219	195	985	225
**Moderately Painful**				
Change Endotracheal Tube	1	1	4	3
Endotracheal Tube Adjust	3	0	5	2
Lumbar Puncture	5	5	10	6
Remove Endotracheal Tube	2	1	19	6
Change Umbilical Dressing	4	4	23	17
Endotracheal Tube Insertion	2	1	30	11
Remove Umbilical Dressing	2	2	37	21
Suction	178	175	351	94
Per Rectal Exam	0	0	1	0
Urinary Catheterization	0	0	1	0
Total Moderately Painful Procedures	197	189	481	160
Total Procedures	2121	2075	11636	2037

Supplementary Table 2. Procedure classification and distribution over first seven days of admission. Data representing the 40 retrospective neonatal file audits from the second study phase.

Name Of Procedure	Day1	Day2	Day3	Day4	Day5	Day6	Day7	Total
**Stressful/Disturbing**								
Physical Exam Frequency	257	186	211	188	178	171	149	1340
Physical Exam Pain Relief Noted	62	37	53	37	46	3	7	245
Position Change Frequency	194	340	345	340	329	331	311	2190
Position Change Pain Relief Noted	43	57	75	74	47	8	12	316
Non Invasive Blood Pressure Frequency	79	135	107	89	71	75	70	626
Non Invasive Blood Pressure Pain Relief Noted	33	43	57	41	33	8	8	223
Temperature Frequency	153	290	292	304	303	307	276	1925
Temperature Pain Relief Noted	36	52	65	57	45	8	10	273
Abdominal Girth Frequency	144	281	288	300	292	300	283	1888
Abdominal Girth Pain Relief Noted	36	51	62	60	40	8	10	267
Diaper Change Frequency	110	267	294	298	283	291	252	1795
Diaper Change Pain Relief Noted	20	43	67	62	44	8	10	254
Weight Frequency	42	41	40	40	40	40	40	283
Weight Pain Relief Noted	7	7	7	7	5	0	1	34
Nasogastric Tube Aspiration Frequency	1	1	1	0	0	1	3	7
Nasogastric Tube Aspiration Pain Relief Noted	1	1	0	0	0	0	0	2
Bedside Chest X-Ray Frequency	10	8	1	7	2	2	2	32
Bedside Chest X-Ray Pain Relief Noted	5	3	1	4	1	1	0	15
Standing X-Ray Frequency	5	2	0	4	1	1	2	15
Standing X-Ray Pain Relief Noted	1	0	0	2	0	0	0	3
Echocardiography Frequency	1	2	1	0	2	0	0	6
Echocardiography Pain Relief Noted	1	0	0	0	0	0	0	1
Ultrasonography Frequency	1	5	4	2	2	1	0	15
Ultrasonography Pain Relief Noted	0	2	1	1	0	0	0	4
Remove Ink Footprint Frequency	6	0	0	0	0	0	0	6
Remove Ink Footprint Pain Relief Noted	1	0	0	0	0	0	0	1
Blood Transfusion Frequency	8	1	0	6	4	1	1	21
Blood Transfusion Pain Relief Noted	4	1	0	3	0	0	0	8
Gastric Lavage Frequency	1	2	0	0	0	1	0	4
Gastric Lavage Pain Relief Noted	0	2	0	0	0	0	0	2
Eliciting Activity Frequency	3	0	0	0	0	0	0	3
Eliciting Activity Pain Relief Noted	3	0	0	0	0	0	0	3

Nebulizer Frequency	3	0	0	0	1	0	4	8
Nebulizer Pain Relief Noted	0	0	0	0	0	0	0	0
Surfactant Administration Frequency	2	4	0	0	0	0	0	6
Surfactant Administration Pain Relief Noted	1	0	0	0	0	0	0	1
**Total Frequency Stressful Procedures**	1020	1565	1584	1578	1508	1522	1393	10170
**Total Frequency Pain Relief Noted For Stressful Procedures**	254	299	388	348	261	44	58	1652
**Mildly Painful**								
Heel Prick Frequency	115	111	94	89	79	71	68	627
Heel Prick Pain Relief Noted	24	30	29	25	18	2	3	131
Venepuncture Frequency	18	13	7	9	8	3	4	62
Venepuncture Pain Relief Noted	4	4	4	2	2	0	0	16
Change IV Frequency	0	4	3	3	2	2	0	14
Change IV Pain Relief Noted	0	2	2	2	0	0	0	6
IV Removal Frequency	0	5	3	5	2	3	1	19
IV Removal Pain Relief Noted	0	3	2	2	0	0	0	7
Blood Sample Frequency	35	25	27	22	15	10	9	143
Blood Sample Pain Relief Noted	12	8	12	8	2	0	0	42
Umbilical Catheterization Frequency	18	3	1	3	0	0	0	25
Umbilical Catheterization Pain Relief Noted	8	1	0	1	0	0	0	10
Nasogastric Tube Insertion Frequency	1	1	2	0	0	1	0	5
Nasogastric Tube Insertion Pain Relief Noted	0	0	2	0	0	0	0	2
Intramuscular Injection Frequency	2	0	0	0	0	0	0	2
Intramuscular Injection Pain Relief Noted	0	0	0	0	0	0	0	0
IV Adjust Frequency	0	0	0	0	3	0	0	3
IV Adjust Pain Relief Noted	0	0	0	0	0	0	0	0
Remove Umbilical Line Frequency	2	2	3	4	4	2	2	19
Remove Umbilical Line Pain Relief Noted	2	1	0	1	2	0	0	6
Nasogastric Tube Adjust Frequency	1	1	0	0	0	0	0	2
Nasogastric Tube Adjust Pain Relief Noted	0	0	0	0	0	0	0	0
Nasogastric Tube Removal Frequency	0	0	1	0	0	1	0	2
Nasogastric Tube Removal Pain Relief Noted	0	0	1	0	0	0	0	1
Eye Drops Frequency	4	5	8	14	13	8	10	62
Eye Drops Pain Relief Noted	0	0	0	4	0	0	0	4
**Total Frequency Mildly Painful Procedures**	196	170	149	149	126	101	94	985
**Total Frequency Pain Relief Noted For Mildly Painful Procedures**	50	49	52	45	24	2	3	225

**Moderately Painful**								
Suction Frequency	70	63	52	47	42	41	36	351
Suction Pain Relief Noted	19	13	25	19	13	3	2	94
Endotracheal Tube Insertion Frequency	20	3	0	3	0	3	1	30
Endotracheal Tube Insertion Pain Relief Noted	6	3	0	1	0	1	0	11
Change Endotracheal Tube Frequency	2	1	0	0	0	1	0	4
Change Endotracheal Tube Pain Relief Noted	1	1	0	0	0	1	0	3
Lumbar Puncture Frequency	1	2	2	5	0	0	0	10
Lumbar Puncture Pain Relief Noted	1	1	1	3	0	0	0	6
Remove Umbilical Dressing Frequency	7	6	5	6	5	6	2	37
Remove Umbilical Dressing Pain Relief Noted	7	5	2	2	2	3	0	21
Change Umbilical Dressing Frequency	5	4	3	4	3	4	0	23
Change Umbilical Dressing Pain Relief Noted	5	3	2	2	2	3	0	17
Endotracheal Tube Adjust Frequency	2	1	0	1	0	1	0	5
Endotracheal Tube Adjust Pain Relief Noted	0	1	0	0	0	1	0	2
Remove Endotracheal Tube Frequency	7	3	2	3	0	4	0	19
Remove Endotracheal Tube Pain Relief Noted	2	1	0	2	0	1	0	6
Per Rectal Exam Frequency	0	0	0	0	0	1	0	1
Per Rectal Exam Pain Relief Noted	0	0	0	0	0	0	0	0
Urethral Catheterization Frequency	0	0	0	0	0	0	1	1
Urethral Catheterization Pain Relief Noted	0	0	0	0	0	0	0	0
**Total Frequency Moderately Painful Procedures**	114	83	64	69	50	61	40	481
**Total Frequency Pain Relief Noted For Moderately Painful Procedures**	41	28	30	29	17	13	2	160
**Total Frequency All Painful Procedures**	310	253	213	218	176	162	134	1466
**Total Frequency Pain Relief Noted For All Painful Procedures**	91	77	82	74	41	15	5	385
**Total Frequency All Painful And Stressful Procedures**	1330	1818	1797	1796	1684	1684	1527	11636
**Total Frequency Pain Relief Noted For All Painful And Stressful Procedures**	345	376	470	422	302	59	63	2037

Supplementary Table 3. Associated conditions neonates developed/presented with at the time of admission. "Pain relief" neonates are those receiving pharmaceutical agents for at least one procedure. "Non pain relief" neonates have not received any pharmaceutical pain relief/central nervous system depressing agents.

	29 Observed Neonates			40 File Audits			All 69 Neonates		
**Associated Conditions**	Pain Relief	Non Pain Relief	Total	Pain Relief	Non Pain Relief	Total	Pain Relief	Non Pain Relief	Total
**Birth Asphyxia**	3	0	3	6	2	8	9	2	11
**Early Onset Sepsis**	8	13	21	7	14	21	15	27	42
**Late Onset Sepsis**	0	0	0	2	1	3	2	1	3
**Hypoxic Ischemic Encephalopathy**	3	0	3	4	1	5	7	1	8
**Apnea of Prematurity**	2	3	5	1	0	1	3	3	6
**Microcephaly**	0	1	1	0	1	1	0	2	2
**Perinatal Depression**	0	0	0	0	3	3	0	3	3
**On Ventilatory Support**	8	6	14	7	4	11	15	10	25
**On Continuous Positive Airway Pressure (CPAP)**	5	5	10	3	6	9	8	11	19
**Congenital Heart Disease**	1	1	2	4	0	4	5	1	6
**Meconium Stained Liquor**	2	2	4	5	6	11	7	8	15
**Meconium Aspiration Syndrome**	1	0	1	2	2	4	3	2	5
**Respiratory Distress Syndrome**	5	7	12	6	8	14	11	15	26
**Electrolyte Imbalance**	1	0	1	2	0	2	3	0	3
**Hyperbilirubinemia**	4	3	7	0	1	1	4	4	8
**Pulmonary Hypertension**	0	0	0	1	0	1	1	0	1
**Pneumonia**	0	1	1	0	1	1	0	2	2
**Septicemic Shock**	0	0	0	1	0	1	1	0	1
**Septicemia with Septic Shock**	0	0	0	0	1	1	0	1	1

**Septic Shock with Multi Organ Damage**	0	0	0	1	0	1	1	0	1
**Cardiogenic Shock**	0	0	0	1	0	1	1	0	1
**Acute Kidney Injury**	0	0	0	1	0	1	1	0	1
**Chronic Lung Disease**	0	0	0	1	0	1	1	0	1
**Deranged Coag Profile**	0	0	0	1	0	1	1	0	1
**Disseminated Intravascular Coagulation**	0	0	0	1	0	1	1	0	1
**Transient Tachypnea of Newborn**	0	0	0	0	1	1	0	1	1
**Exchange Transfusion Required**	0	0	0	1	1	2	1	1	2
**Surfactant Required**	2	0	2	1	1	2	3	1	4
**Anemia of Prematurity**	0	2	2	0	0	0	0	2	2
**Hypoglycemia**	2	1	3	0	0	0	2	1	3
**Pneumothorax**	1	2	3	0	0	0	1	2	3
**Meningitis**	2	0	2	0	0	0	2	0	2
